# Objective measurement of forward-scattered light in the human eye: An electrophysiological approach

**DOI:** 10.1371/journal.pone.0214850

**Published:** 2019-04-04

**Authors:** Benjamin Solf, Stefan Schramm, Dietmar Link, Sascha Klee

**Affiliations:** Institute of Biomedical Engineering and Informatics, Technische Universität Ilmenau, Ilmenau, Germany; University of Rochester Medical Center, UNITED STATES

## Abstract

**Purpose:**

Psychophysical measurements are used to examine the perception of ocular stray light, for example, with C-Quant. These measurements are subjective due to their principles. This work aims to determine ocular stray light objectively; thus, a psychophysical method is transferred into an electrophysiological setup.

**Methods:**

Stray light perception was measured using steady-state visual evoked potentials (VEPs) in 10 healthy subjects (7 males, 3 females, mean age ± SD: 29.6 ± 4.1 years). Stray light emulating filters (Tiffen Black Pro Mist 2) were used for simulating the effect of cataracts to validate the results for increased scattered light conditions. Based on the direct compensation method, the stimulus consisted of a central test field (radius = 2°) with a luminance adjustable compensation light and surrounding ring-shaped stray light source (radius = 5 to 10°). Both flickered in the counter phase at a frequency of 7.5 Hz. The stimuli were presented for 15 luminance levels of the compensation light. The recorded steady-state VEPs at Oz channel were transformed by means of Fourier analysis. The magnitudes at the evoked frequency were plotted against the measured brightness levels of the compensation light. By fitting two linear functions to the resulting data points, a robust minimum log(L_eq_) was determined, which was correlated with the amount of stray light perception. We measured the stray light parameter log(s_c_) using C-Quant. For comparison, our results were converted into the C-Quant equivalent parameter log(s_epm_) and paired t-tests were performed for normal distributed results.

**Results:**

A significant difference is observed between log(s_epm_) (without filter) and log(s_epm_) (with BPM 2 Filter) (p>0.05). No significant difference is observed between log(s_epm_) (without filter) and log(s_c_) (without filter) (p > 0.05) and between log(s_epm_) (with BPM 2 filter) and log(s_c_) (with BPM 2 filter) (p > 0.05).

**Conclusion:**

The electrophysiological approach offers the ability to measure stray light perception in an objective manner.

## Introduction

Retinal image quality is reduced in the presence of ocular stray light. Increased stray light and its perception lead to glare phenomena, resulting in vision impairment. This effect is caused by a veiling luminance on the retina, which is added to the image, forming light. In 1939, Stiles and Holladay proposed the concept of an equivalent veiling luminance L_eq_, induced by a glare source E_gl_ at a defined stray angle [1]. Based on this concept, various improvements and adaptations for different ages and glare angles were reported [2]. Common approaches for evaluating ocular stray light include the direct compensation method (DCM) [3] and the compensation comparison method (CCM) [4]. These psychophysical methods use the subject perception of forward-scattered intraocular light. The CCM is implemented in the commercial product C-Quant (OCULUS Optikgeräte GmbH, Wetzlar, Germany) and is the gold standard for assessing retinal stray light perception. Due to its principle, the stray light measured by C-Quant is a subjective representation, with various drawbacks, such as a strong dependence on subject cooperation, as well as high variability [5, 6].

In this study, we aimed to analyze an objective determination of stray light perception caused by forward-scattered light in the human eye. Therefore, we transferred a psychophysical approach to an electrophysiological measurement environment in order to combine the benefits of psychophysical methods with the objective characteristics of electrophysiological measurements. Thus, we used an adapted setup based on the DCM for stimulating and recording the steady-state visual evoked potentials (VEPs) of the subject.

## Materials and methods

### Subjects

A total of 10 healthy Caucasian subjects (7 males, 3 females, age, 24 to 36 years) were examined. The subjects have given written informed consent to publish these case details, and the study was approved by the Ethics Committee of the Faculty of Medicine of the Friedrich Schiller University Jena and conducted in compliance with the Declaration of Helsinki. Sphere (-2.25 to +1.25 dpt) and astigmatism (-1.0 to 0 dpt) were measured using an autorefractor (CX 1000, Rodenstock Instruments, Erlangen, Germany).

### Measurement setup

**Fig 1:**
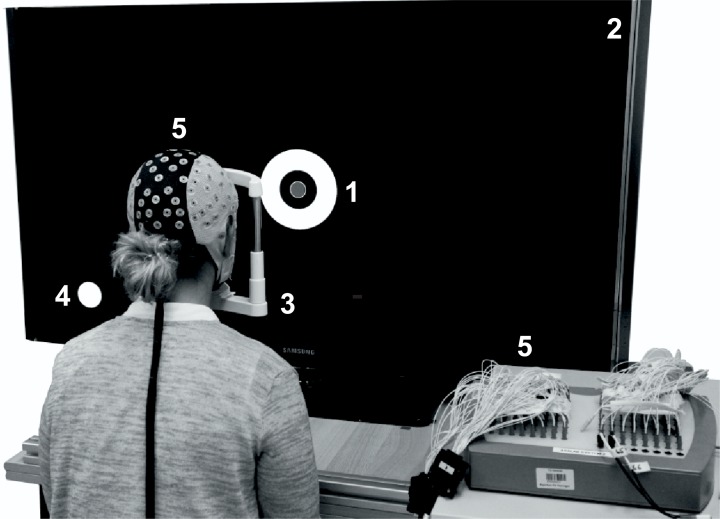
Stimulation setup with stimulus used (1). The stimulator (2) was a 52-inch LCD with full-array LED-backlight illumination. The subject head was placed in a chin-forehead rest to enable a constant position. The stimulation and measurement setups were synchronized by a PIN photodiode placed on the synchronization field (3) in the lower left corner of the stimulator. The EEG was recorded using a 64-channel waveguard EEG system (4).

A 52-inch (LE-52F9BD, Samsung Corp, Seoul, South Korea) full-array LED-backlight liquid crystal display (LCD) with a native resolution of 1920 x 1080 pixels, maximum luminance of 350 cd/m^2^, and refresh rate of 60 Hz was used as the stimulator. The luminance stability was proven using a spectroradiometer (specbos 1201, JETI Technische Instrumente GmbH, Jena, Germany), and the frequency stability using a PIN photodiode (BPX 65, Siemens, Munich, Germany) and oscilloscope (TDS 3054, Tektronix Inc., Beaverton, USA). Stimuli were generated by means of self-developed stimulation software. The steady-state VEPs were recorded with a 64-channel EEG system (waveguard original, ANT Neuro, Enschede, Netherlands). In order to synchronize the stimulation with the EEG recording, we used a PIN photodiode placed in the lower left corner of the stimulator, connected to a bipolar channel of the EEG system. The subjects were placed in front of the stimulator with their heads fixated in a chin-forehead rest to ensure a central and constant eye position at a distance of 50 cm, as well as to suppress motion artifacts (Fig 1).

### Stimulation and perception

**Fig 2:**
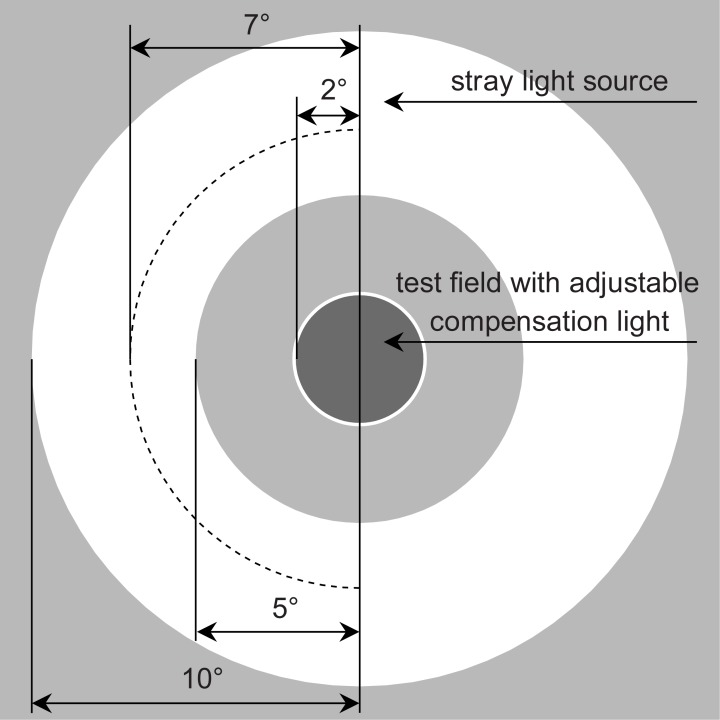
Illustration of stimulus used. The stimulus consisted of a test field (radius r = 2°) with an adjustable compensation light surrounded by a ring-shaped stray light source (radius r = 5 to 10°).

Fig 2 illustrates the stimulus used, which consisted of a central test field with an adjustable compensation light (radius r = 2°) surrounded by a ring-shaped stray light source. The stray light source had an inner radius of θ_i_ = 5° and outer radius of θ_o_ = 10°, resulting in a stray light angle of 7° [7].

**Fig 3:**
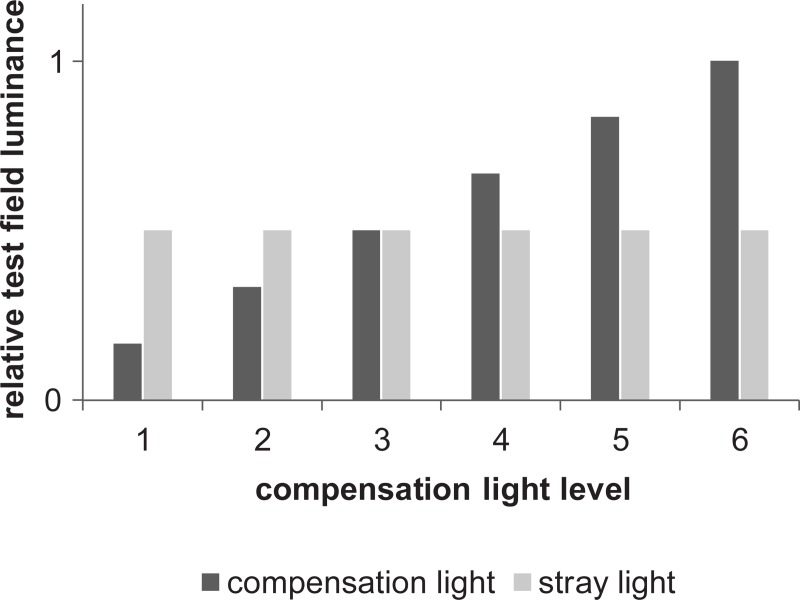
Resulting test field luminance, dependent on compensation light level. The stray light induced by the stray light source was a constant amount. By varying the luminance of the compensation light, the modulation depth in the test field changed. At the full compensation point (compensation light level 3), the compensation light was equal to the induced stray light.

During the stimulation, the stray light source appeared periodically and induced a flicker effect in the test field. The modulation depth of the induced flicker effect was dependent on the subject individual stray light perception. The compensation light was added in the counter phase. By varying the compensation light luminance, the modulation depth in the test field changed. At the full compensation point, the equivalent veiling luminance caused by the stray light perception was equal to the luminance caused by the compensation light. The flicker effect in the test field disappeared (Fig 3).

The stray light source had a maximum luminance of E_gl_ = 350 cd/m^2^. The flicker frequency was set to 7.5 Hz, as subjects exhibit effective flicker perception at between 5 and 10 Hz [8–11], and in respect to the stimulator refresh rate. Due to the stimulation frequency being above 3 Hz, the recorded responses were steady-state responses, which contained components at the stimulation frequency and their harmonics [12, 13].

A total of 15 compensation light levels L_cl_ were presented within the range of 0.9 to 9.4 cd/m^2^. Each level was presented for 10 s, followed by a resting period of approximately 5 s, resulting in a total measurement time of around 4 min. To validate the method for increased stray light conditions, stray light emulating filters were used (Tiffen Black Pro Mist—BPM 2, The Tiffen Company, Hauppauge, New York, USA) [4, 14–16]. Therefore, compensations light levels L_cl_ within the range of 2.2 to 29.5 cd/m^2^ were presented. The compensation light level sequences were randomized. All stimulations were performed binocularly at an average room luminance (four walls and ceiling) of about 1 cd/m^2^. The illuminance at the eyes was 30 lx (stray light source on). Moreover, we determined the individual alpha frequencies of the subjects in order to avoid the resonance and entrainment effects that emerge in the alpha band [17–19].

**Fig 4:**
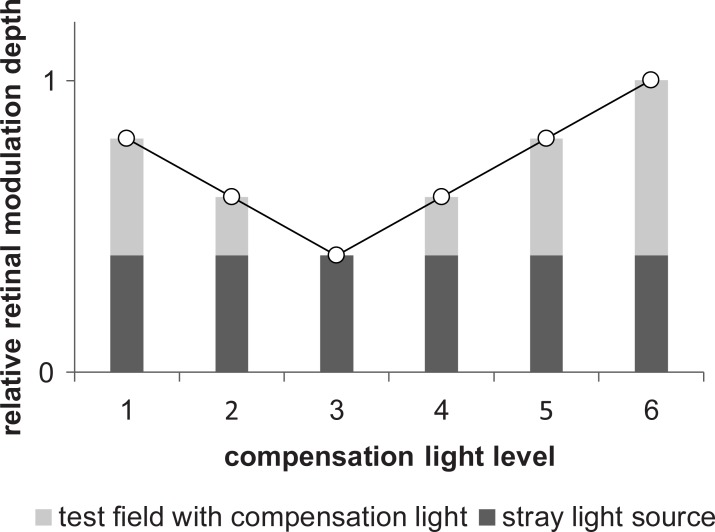
Resulting retinal modulation depth for different amounts of compensation light. The stray light source applied a constant retinal modulation depth to the extrafoveal area. This led to constant stimulation for the different compensation light levels. The retinal modulation depth of the test field is dependent on the level of stray light perception and adjustable compensation light. Due to the compensation processes, the modulation depth of the test field is zero at the full compensation point (compensation light level 3). The retinal modulation depth is only applied by the stray light source.

The subject fixation on the test field led to stimulation of the extrafoveal area by the stray light source and foveal stimulation by the test field. Due to the constant luminance level, the stray light source induced a constant amount of retinal light in the extrafoveal area. The test field modulation depth was dependent on the subject stray light perception and compensation light luminance, resulting in a variable retinal light amount. Thus, the extrafoveal stimulation by the stray light source evoked a constant magnitude at the corresponding frequency. The magnitude evoked by the test field was dependent on the modulation depth. At the full compensation point, the resulting electrophysiological response was only evoked by the stray light source. We assumed a correlation between the full compensation point and compensation light luminance level that evoked the minimum response (Fig 4).

In order to compare our results, ocular stray light was measured with C-Quant. The subjects looked at a two-parted test field surrounded by a flickering stray light source. The stray light perception caused a flicker effect in the test field. A compensation light was added to one test field half to reduce the flicker effect. The subject’s task was to choose the test field half showing the greatest flicker effect. Based on the responses of the subject, a psychophysical curve was generated which describes the stray light perception of the subject. For each subjects, 12 measurements were performed consisting of six measurements without filter and six with BPM 2 filter (three per eye). We calculated the mean value ± SD for the resulting stray light parameters log(s_c_) without filter and with BPM 2 filter per eye and the mean values for log(s_c_) without filter and with BPM 2 filter in order to obtain a binocular stray light value [20].

### Recording and preprocessing

**Fig 5:**
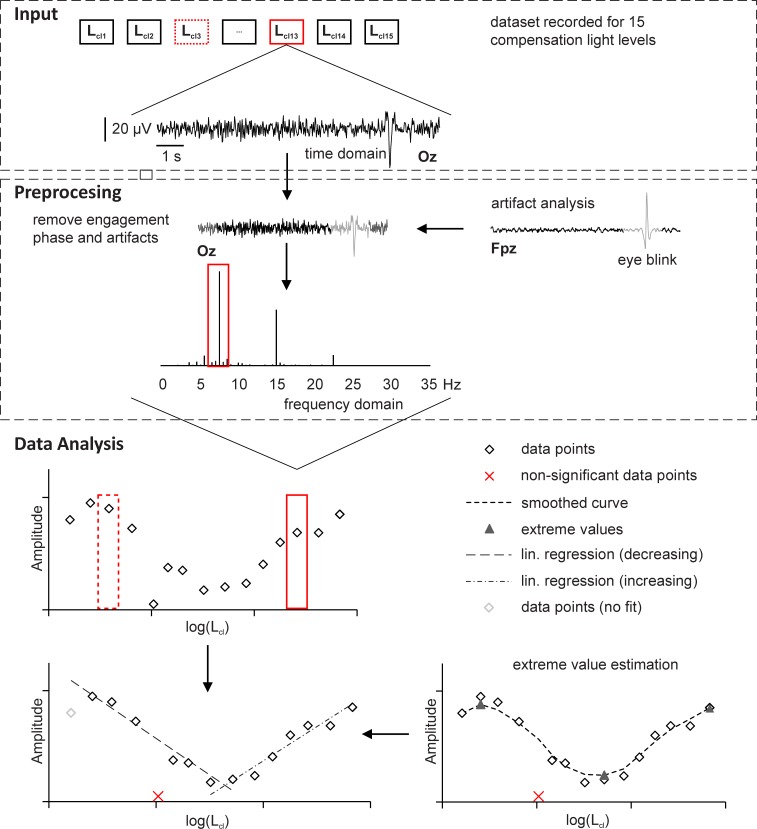
Schematic of the signal processing. Input: The input data consist of a dataset recorded for 15 compensation light levels with a length of 10 s per trail (Oz channel). Preprocessing: The first and final second were excluded from each trail to reduce the engagement effect and the remaining trail was separated into four sections. Eye blink artifacts were detected in the FPz channel and artifact-affected parts of the trail were excluded from further analysis. The remaining parts were averaged and transformed in the frequency domain. Data analysis: The amplitudes at the stimulation frequency (data points) were plotted against the measured compensation light levels log(L_cl_). Non-significant data points were excluded and the remaining points were smoothed with a moving average (smoothed curve). The global minimum and two local maxima (extreme values) were determined as starting points for two linear regressions (lin. regression (decreasing) and lin. regression (increasing)). Thereby, the influence of non-causal data points (data points (no fit)) was reduced. The intersection of the regressions correlates with the full compensation point.

The EEG was recorded using a 64-channel EEG cap with Ag/AgCl electrodes (10–10 system) (Fig 1). The amplifier used a common average reference and the ground electrode was placed at AFz. A sample rate of 1024 Hz was used. The recorded data were digitally bandpass filtered with a zero-phase Butterworth filter of order 4, with cut-off frequencies at 2 and 35 Hz. The data recorded from the Oz channel were used. The first and the last second of each 10 s trail were excluded in order to reduce the influence of the engagement phase on the intermittent stimuli [17]. Each trail was separated into four 2 s sections. Artifact analysis was performed in the FPz channel to detect eye blinks and concerning sections were rejected from further analysis. The remaining sections were averaged and transformed in the frequency domain using Fourier transformation (Fig 5). Therefore, an integer number of stimulus periods was used to avoid frequency overspill [21].

### Data analysis and statistics

The data analysis was performed using MATLAB (MathWorks Inc., Natick, USA). Due to the test field modulation depth near the visual perceptional threshold, the significance of the magnitude at the evoked frequency for each compensation light level was determined. Therefore, the next higher and lower magnitudes to the evoked frequency were used as noise estimation [22, 23]. Non-significant responses (p > 0.05) were excluded from further analysis.

The magnitudes at the evoked frequency were plotted against the measured logarithmic luminance levels log(L_cl_) of the compensation light. The data points were filtered using a moving average, with a window size of 3 data points, and the global minimum and two local maxima were determined. Starting from the minimum estimation, the curve was separated into two parts. Each part was approximated by means of linear regression, based on the data points included by the local maximum and global minimum (Fig 5). Thus, the influence of non-causal data points was minimized. The resulting intersection point of the two linear functions represents the robust minimum, and accordingly, the equivalent veiling luminance log(L_eq_). The log(L_eq_) value was converted into a C-Quant equivalent parameter log(s_epm_) using the formula in Eq 1. Detailed information is provided in [7].

$$s_{epm}(\theta )=L_{eq}∙{\left(2\pi ∙{(\frac{\pi }{180})}^{2}∙ln(10)∙E_{gl}∙log\left(\frac{\theta_{o}}{\theta_{i}}\right)\right)}^{-1}$$ Eq 1

SPSS Statistics 24 (IBM, Armonk, USA) was used for the statistical analysis. The distributions of log(s_c_) and log(s_epm_) (without filter and with BPM 2 filter) were determined using the Shapiro-Wilk test [24]. Log(s_epm_) without filter and log(s_epm_) with BPM 2 filter were compared in case of normal distributed results by paired t-test and for non-normal distributed results by Wilcoxon rank-sum test [25, 26]. In order to compare the results with the gold standard, the paired t-test was performed for the normal distributed results (p > 0.05), while the Wilcoxon rank-sum test was used for the non-normal distributed results (p < 0.05). Furthermore, Bland-Altman analysis [27, 28] was performed.

## Results

**Fig 6:**
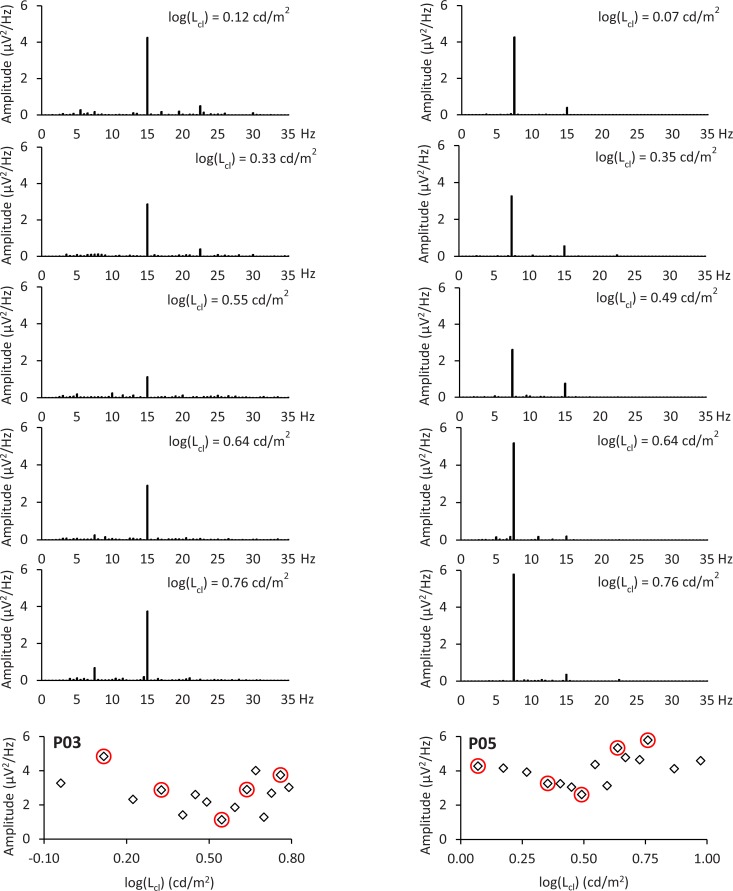
VEPs in the frequency domain for two subjects. The VEPs of 5 data points (highlighted in red) are shown for two subjects (P03: log(L_cl_) = 0.12, 0.33, 0.55, 0.64 and 0.76 cd/m^2^; P05: log(L_cl_) = 0.07, 0.35, 0.49, 0.64 and 0.76 cd/m^2^). The major response for subject P03 is at 15 Hz and for subject P05 at 7.5 Hz.

**Fig 7:**
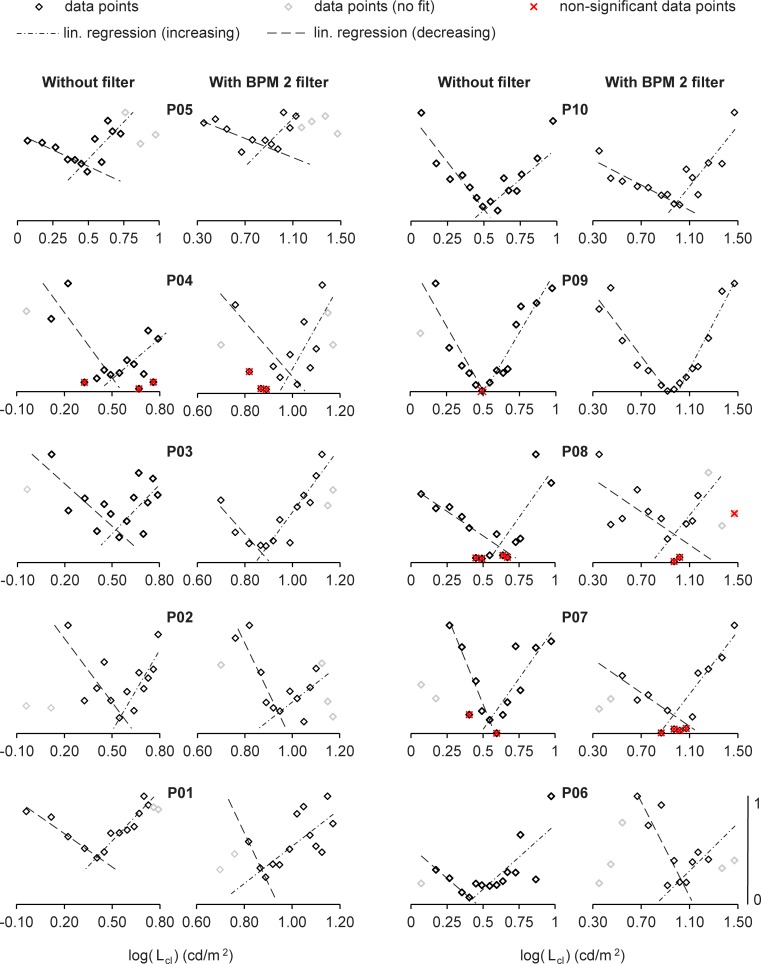
Normalized measured data points with estimated full compensation point for 10 subjects. The data points were plotted against the measured compensation light levels log(L_cl_). All data points exhibit the expected course. Non-significant data points were determined for subjects P04, P07, P08, and P09.

In Fig 6, the VEPs in the frequency domain of two subjects are shown for 5 data points each. The evoked major response for subject P03 is at 15 Hz and the major response for subject P05 is at 7.5 Hz. The results of the electrophysiological measurements of the 10 subjects are illustrated in Fig 7. The data points were normalized to the maximum value of each subject, and all data points exhibit the expected course. For six of the 10 subjects, all measured data points were significant in relation to the noise estimation of the neighboring frequencies. Non-significant data points occurred for four subjects. The results of the C-Quant measurements are shown in Table 1. The determined values for log(L_eq_), log(s_epm_), and log(s_c_) are summarized in Table 2. The values for log(s_epm_) (without filter), log(s_c_) (without filter), and log(s_c_) (with BPM 2 filter) are normal distributed (p > 0.05). The values for log(s_epm_) (with BPM 2 filter) are non-normal distributed (p<0.05). A significant difference is observed between log(s_epm_) (without filter) and log(s_epm_) (with BPM 2 Filter) (p>0.05). No significant difference is observed between log(s_epm_) (without filter) and log(s_c_) (without filter) (p > 0.05) and between log(s_epm_) (with BPM 2 filter) and log(s_c_) (with BPM 2 filter) (p > 0.05).

**Table 1:** Mean monocular stray light parameter log(s_c_) and standard deviation of the C-Quant measurements without and with BPM 2 filter.

	Without filter				With BPM 2 filter			
	Left eye		Right eye		Left eye		Right eye	
**Subject**	log(s_c_)	SD	log(s_c_)	SD	log(s_c_)	SD	log(s_c_)	SD
P01	0.75	0.01	0.73	0.02	1.20	0.01	1.21	0.02
P02	0.87	0.03	0.90	0.02	1.27	0.02	1.29	0.01
P03	0.88	0.05	0.83	0.05	1.22	0.04	1.21	0.03
P04	0.80	0.03	0.83	0.05	1.30	0.03	1.30	0.03
P05	0.81	0.01	0.81	0.05	1.23	0.04	1.21	0.05
P06	0.79	0.03	0.78	0.03	1.37	0.08	1.35	0.07
P07	0.89	0.05	0.83	0.02	1.32	0.02	1.29	0.05
P08	0.92	0.03	0.94	0.09	1.28	0.02	1.31	0.02
P09	0.84	0.01	0.85	0.02	1.30	0.03	1.29	0.01
P10	0.86	0.02	0.85	0.04	1.33	0.01	1.31	0.05

**Table 2:** Equivalent veiling luminance log(L_eq_), corresponding stray light parameter log(s_epm_), and stray light parameter log(s_c_) determined by C-Quant measurement of 10 subjects.

	Electrophysiological measurement				C-Quant	
	Without filter		With BPM 2 filter		Without filter	With BPM 2 filter
**Subject**	log(L_eq_) (cd/m^2^)	log(s_epm_)	log(L_eq_) (cd/m^2^)	log(s_epm_)	log(s_c_)	log(s_c_)
P01	0.41	0.74	0.87	1.20	0.74	1.21
P02	0.56	0.89	0.94	1.27	0.89	1.28
P03	0.53	0.86	0.87	1.20	0.86	1.22
P04	0.50	0.83	0.99	1.32	0.82	1.30
P05	0.47	0.80	0.87	1.20	0.81	1.22
P06	0.42	0.76	1.02	1.35	0.79	1.36
P07	0.54	0.88	0.97	1.30	0.86	1.31
P08	0.61	0.94	0.97	1.30	0.93	1.30
P09	0.51	0.84	0.97	1.30	0.85	1.30
P10	0.51	0.84	0.96	1.32	0.86	1.33

**Fig 8:**
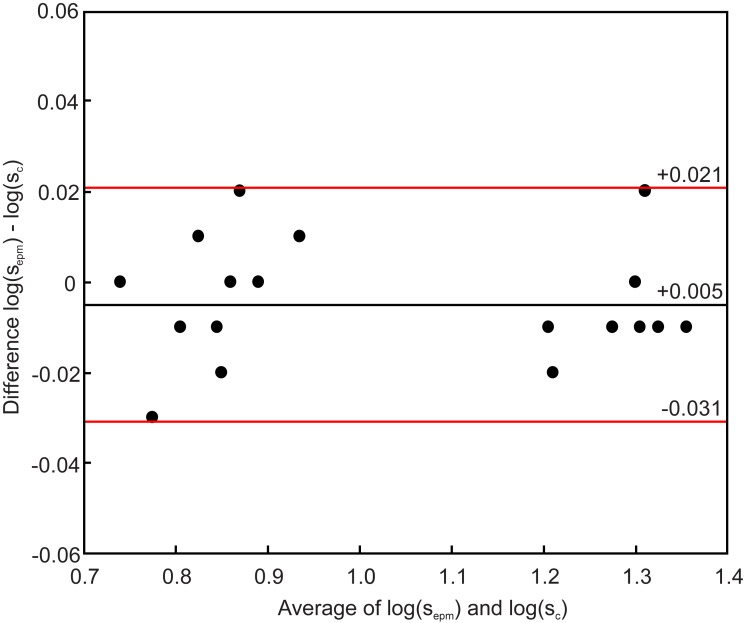
Bland-Altman plot of log(s_epm_) and log(s_c_) for 10 subjects. The mean difference is -0.005 with the limits of agreement of +0.021 to -0.031. All paired data are within the limits of agreement.

Fig 8 illustrates the Bland-Altman plot for log(s_epm_) and log(s_c_). The mean difference is -0.005 with the limits of agreement of +0.021 to -0.031. All paired data are within the limits of agreement.

## Discussion

To the best of our knowledge, this paper presents the first electrophysiological approach for measuring ocular stray light perception. Therefore, steady-state VEPs obtained by stimuli based on the psychophysical DCM were used. By combining the DCM with EEG measurements, we were able to overcome the general disadvantages of subjective measurement methods as well as the limitations of the DCM [4]. In 10 subjects, we determined the stray light perception caused by forward-scattered light, and found no significant difference compared to the gold standard for normal and increased stray light conditions.

The compensation light range was adjusted due to the high stray light values for subjects P02 to P04. For subjects P01 to P04, the compensation light for stimulations without filter was within the range of 0.9 to 6.2 cd/m^2^ and for stimulations with BPM 2 filter within the range of 5.0 to 14.8 cd/m^2^; for subjects P06 to P10, the range for stimulations without filter was 1.2 to 9.2 cd/m^2^ and for stimulations with BPM 2 filter 2.2 to 29.5 cd/m^2^.

Four of the 10 subjects (P04, P07, P08, and P09) exhibited non-significant data points (Fig 7). In subjects P07, P08, and P10, the non-significant data points occurred in the area of the equivalent veiling luminance log(L_eq_). A possible reason for this is the low modulation depth in this area, resulting in a low signal-to-noise ratio. However, the veiling luminance log(L_eq_) was determined in all subjects. With a closed-loop system, non-significant responses can be identified and the corresponding compensation light levels can be repeated online; making the determination of log(L_eq_) more robust.

By means of stimulation with intermittent stimuli, the evoked response contains frequency components related to the stimulation frequency and its harmonics, and the main component is expected at the stimulation frequency. In our investigations, five subjects exhibited the main component at the stimulation frequency (7.5 Hz), and five at the first harmonic (15 Hz). This behavior has also been reported in clinical trials [29]. The reason for this may be found in the latency differences leading to an increased magnitude in the first harmonic [30]. In our study, the latency difference was caused by the spatial distribution of the extrafoveal stray light source and foveal test field [31, 32]. However, the origin of the harmonic frequencies is not fully understood and is probably due to nonlinear effects in the visual system [33–35].

The prevalence of cataracts increases with increasing age [36, 37]. In contrast, the individual alpha frequency decreases [38]. Salchow et al. [17] reported entrainment effects within the frequency range of 0.9 to 1.1*individual alpha frequency as well as in the range of 0.4 to 0.55*individual alpha. In our study, the subjects were aged from 22 to 36 years. Their individual alpha frequencies were within the range of 8.9 to 12 Hz. Thus, resonance and entrainment effects should not have had any impact. For potential cataract grading use, the resonance and entrainment may be relevant.

Instructions to avoid eye movements or blinks can lead to unwanted brain activity [39, 40]. Eye blinks occurred due to the stimulation period of 10 s. State-of-the-art approaches in artifact rejection include independent component analysis (ICA)-based methods [41]. However, following artifact rejection using ICA there still remain artifactual components in the signal [42]. As we performed stimulations near the perception threshold, we detected and excluded trials of concern from further analysis.

Owing to the low test field modulation depth, the bright stray light source is a potential distraction during fixation. Small deviations (≤ 2°) are not relevant to the evoked responses, as a homogeneous light distribution of the test field can be assumed. In contrast, fixation on the stray light source results in adaption processes, affecting the following measurement steps. Thus, non-significant signal responses arise from a low signal-to-noise ratio. Accordingly, fixation control should be considered in future investigations.

A factor associated with psychophysical methods is the need for subject interaction, which may be challenging for subjects. The new approach offers an improvement for subjects who had trouble with the assessing the flicker intensity in the gold standard. Moreover, the interaction provides potential uncertainty and may lead to manipulation of the results. C-Quant uses a quality parameter to reject unreliable measurements [43]. Our proposed approach overcomes this drawback by excluding subject interaction. Thus, we were able to determine the stray light perception of the forward-scattered light objective. Our approach can be compared to other objective methods, such as Hartmann-Shack aberrometry as well as double-pass imaging-based methods [44–46]. These methods determine the amount of stray light on the retinal layer. However, stray light perception is not based solely on retinal stray light [1, 47]. Our approach combines the benefits of psychophysical measurement methods such as the description of the subject perception and the objective characteristics of the Hartmann-Shack or other double-pass-based methods. Nevertheless, each method offers unique advantages for specific purposes.
